# Hydrogen gas and the gut microbiota are potential biomarkers for the development of experimental colitis in mice

**DOI:** 10.1017/gmb.2023.17

**Published:** 2023-11-06

**Authors:** Yuta Fujiki, Takahisa Tanaka, Kyosuke Yakabe, Natsumi Seki, Masahiro Akiyama, Ken Uchida, Yun-Gi Kim

**Affiliations:** 1 Research Center for Drug Discovery, Faculty of Pharmacy and Graduate School of Pharmaceutical Sciences, Keio University, Tokyo 105-8512, Japan; 2 Division of Biochemistry, Faculty of Pharmacy and Graduate School of Pharmaceutical Sciences, Keio University, Tokyo 105-8512, Japan; 3 Department of Materials Engineering, The University of Tokyo, Tokyo 113-8656, Japan

**Keywords:** gut microbiota, hydrogen gas, experimental colitis, inflammatory bowel disease, biomarker

## Abstract

Inflammatory bowel disease (IBD) is a chronic disease characterised by repeated relapses and remissions and a high recurrence rate even after symptom resolution. The primary method for IBD diagnosis is endoscopy; however, this method is expensive, invasive, and cumbersome to use serially. Therefore, more convenient and non-invasive methods for IBD diagnosis are needed. In this study, we aimed to identify biological gas markers for the development of gut inflammation. Using dextran sulphate sodium (DSS)-induced colitis mouse models, five biological gases were analysed to identify predictive markers for the development of gut inflammation. Additionally, the correlation between the changes in gas composition, gut microbiota, and inflammatory markers was assessed. The hydrogen (H_2_) level was found to be negatively correlated with the level of lipocalin-2 (LCN2), a gut inflammation biomarker, and weight loss due to DSS-induced colitis. Furthermore, gut microbes belonging to the Rikenellaceae and Akkermansiaceae families were positively correlated with LCN2 levels and weight loss, whereas Tannerellaceae abundance was negatively correlated with LCN2 level and weight loss and positively correlated with H_2_ levels. This study provides new insights for IBD diagnosis; the H_2_ levels in biological gases are a potential biomarker for intestinal inflammation, and specific gut microbes are associated with H_2_ level changes.

## Introduction

Inflammatory bowel disease (IBD), including ulcerative colitis (UC) and Crohn’s disease (CD), is a chronic inflammatory disease that develops through complex interactions among genetic, immune, environmental, and microbial factors(Zhang and Li, 2014). The incidence of IBD has increased worldwide, posing a significant health, social, and economic burden (Jairath and Feagan, 2020). It is conceivable that earlier interventions in the disease course, potentially during an asymptomatic stage, will be more effective in resetting dysregulated immune pathways and other pathological processes that drive the disease from the preclinical to clinical stages. However, IBD is characterised by relapsing and remitting phases with a high recurrence rate even after symptom resolution (Yamamoto, 2005; Rogler et al., 2013), making it difficult to predict its recurrence (Liverani et al., 2016).

Endoscopy is the gold standard for monitoring patients with IBD; however, the method is time-consuming, expensive, and invasive (Marlicz et al., 2018). Furthermore, endoscopy scores are poorly reproducible and highly dependent on the experience of the endoscopist (Regueiro et al., 2011; Marlicz et al., 2018). Therefore, there is an urgent need to identify more reliable, non-invasive surrogate biomarkers and establish measurement methods to reduce patient burden and costs.

Several biological samples such as serum and faecal inflammatory markers (Oh et al., 2017), serum antibodies (Dubinsky, 2008), and exhaled breath have attracted attention as biomarkers. Serum C-reactive protein (CRP), erythrocyte sedimentation rate (ESR), anti-*Saccharomyces cerevisiae* antibody (ASCA), perinuclear antineutrophil cytoplasmic antibody (pANCA), and faecal calprotectin are employed clinically (Iskandar and Ciorba, 2012; Fengming and Jianbing, 2014). In addition to these inflammation-associated biomarkers, breath gases can be potential biomarkers, given the easy and rapid measurements that allow repeated sampling and the non-invasive nature of their analysis (Das et al., 2016). Previous studies have demonstrated a relationship between IBD and exhaled breath (Hicks et al., 2015; Monasta et al., 2017). For example, ethane and pentane concentrations in the breath of patients with UC and IBD, respectively, were significantly higher than those in healthy individuals (Sedghi et al., 1994; Dryahina et al., 2013). Furthermore, machine learning has been used to distinguish between healthy controls, patients with active CD, and those in remission based on volatile organic compounds (VOCs) (Bodelier et al., 2015). Therefore, breath analysis may be a practical strategy for diagnosing IBD. However, most studies have only focused on volatile compounds that have large molecular weights and are easy to quantify, whereas other molecules including low-molecular-weight compounds have not been assessed as much (Boots et al., 2012; Ratcliffe et al., 2020; Van Malderen et al., 2020). Additionally, human studies are heterogeneous in terms of age, dietary factors, and history of antibiotic and other drug use (Hicks et al., 2015; Henderson et al., 2022). Therefore, the effect of IBD pathology on exhaled breath remains unclear. Furthermore, most studies have focused on patients with active IBD, and using these breath gases as possible predictive biomarkers for the development or recurrence of IBD is yet to be fully elucidated.

Advances in next-generation sequencing and metagenomic analysis technologies have suggested that the intestinal microbiota, an environmental factor, is closely associated with IBD pathogenesis (Caruso et al., 2020; Lavelle and Sokol, 2020). Multiple studies have documented differences in the composition, diversity, and metabolites of the gut microbiota between IBD patients and healthy individuals (Ohkusa and Koido, 2015; Weingarden and Vaughn, 2017). Some metabolites derived from the gut microbiota are absorbed by the host, reach the blood–lung barrier via the bloodstream, and are quickly excreted via the airways as exhaled breath, whereas other metabolites are excreted from the body as faeces and urine (Sharon et al., 2014; Vernocchi et al., 2016). Thus, biological samples, such as exhaled breath, faeces, urine, and sweat, reflect gut microbiota diversity and metabolism, and thus, the abnormal inflammatory processes occurring in the human body (Arasaradnam et al., 2014).

In this study, dextran sulphate sodium (DSS)-induced colitis mouse models were used to identify the predictive biological gas markers for the development of gut inflammation. Changes in five biological gas components, namely hydrogen (H_2)_, ammonia (NH_3)_, hydrogen sulphide (H_2_S), methanethiol (CH_3_SH), and ethanethiol (C_2_H_5_SH), during the development of experimental colitis, were continuously analysed. Furthermore, the composition of the gut microbiota and inflammatory markers over time were examined to assess the correlations between changes in the biological gas composition, gut microbiota, and inflammatory markers.

## Results

### Setup for measuring biogenic gases

**Figure 1.**
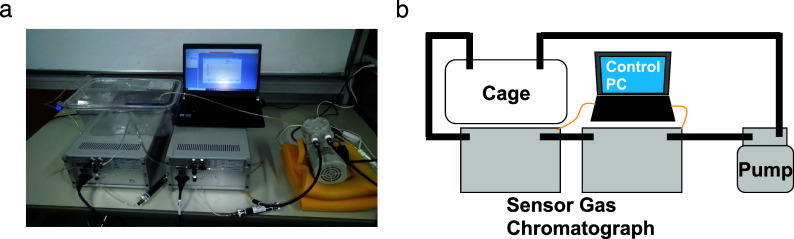
(a) Photograph and (b) schematic of the breath sampling system. The air in the cage was circulated by the pump. Using the sensor gas chromatographs in the circulation path, (H_2_), ammonia (NH_3_), hydrogen sulphide (H_2_S), methanethiol (CH_3_SH), and ethanethiol (C_2_H_5_SH) concentrations were measured. The sensor gas chromatograph consisting of a gas column and a metal oxide semiconductor gas sensor enabled selective and sensitive gas sensing.

Biogenic gases H_2_, NH_3_, H_2_S, CH_3_SH, and C_2_H_5_SH in the cage were sampled and measured using sensor gas chromatographs installed in an air flow line ventilated by a diaphragm pump (Figure 1a,b). The sampling rate of the gas concentration depended on the refresh time for each measurement of the gas column and gas sensor in the sensor gas chromatograph, and the average sampling rate was 10 points/h. The sampled gas concentration reflected the breath and skin gas of the mice and the gas from faeces.

### *H*_*2*_ *fluctuation correlates with intestinal inflammation*

**Figure 2.**
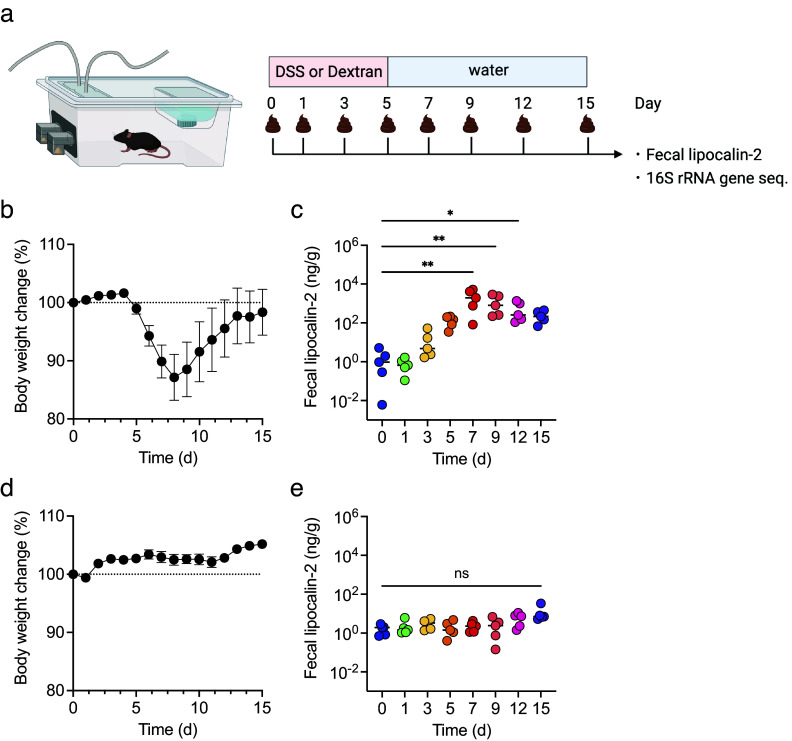
The mice were treated with dextran sulphate sodium (DSS) or dextran for 5 days and monitored for 15 days (n = 5). (a) The experimental design. (b and d) body weight changes in DSS- (b) or dextran-treated mice (d). (c and e) faecal lipocalin-2 (LCN2) levels on days 0, 1, 3, 5, 7, 9, 12, and 15 in DSS- (c) or dextran-treated mice (e). Friedman’s test was used in (c) and (e). *p < 0.05; ** *p* < 0.01; *** *p* < 0.001; ns., not significant. All the experiments were conducted three independent times.

To examine the correlation between inflammatory levels in DSS-induced colitis and changes in biological gas composition, changes in body weight and faecal lipocalin-2 (LCN2) levels were first monitored for 15 days. All mice were treated with DSS or dextran, a DSS analogue, as a negative control (Figure 2a). In DSS-treated mice, body weight decreased from day 5 and was restored by day 9 (Figure 2b). Faecal LCN2 level increased gradually, peaked on day 7, and remained high until day 15 (Figure 2c). In contrast, in dextran-treated mice, body weight did not change dramatically for 15 days, and the faecal LCN2 level was unaltered (Figure 2d,e). These results indicate that DSS, but not dextran, induced body weight loss and increased the levels of faecal inflammatory markers.

**Figure 3.**
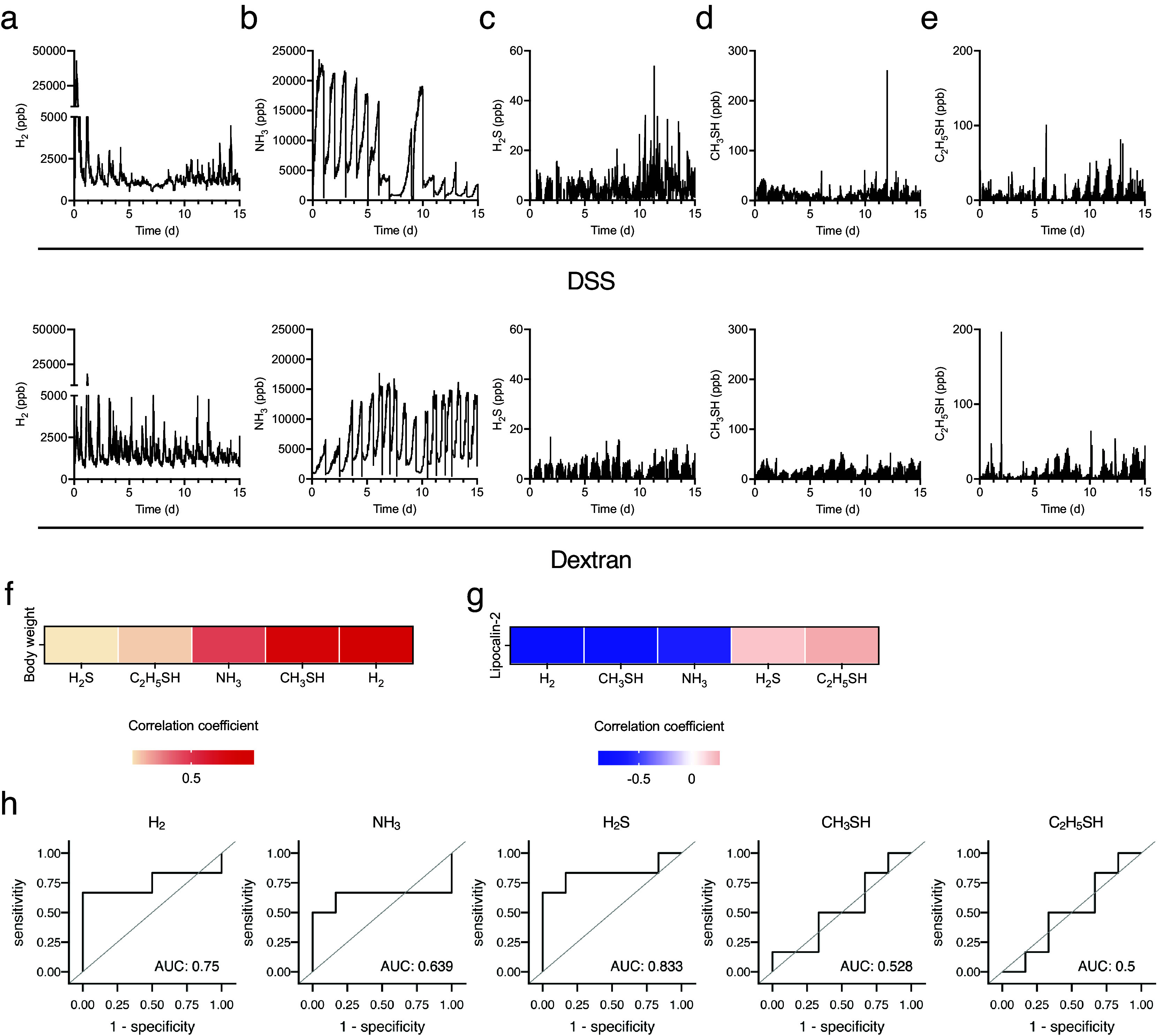
Five biogenic gases were detected using the instrument introduced in Figure 1 for 15 days. (a–e) The kinetics of (a) H_2_, (b) NH_3_, (c) H_2_S, (d) CH_3_SH, and (e) C_2_H_5_SH for 15 days. (f and g) The heatmap of correlation between the average body weight change (f) or level of faecal LCN2 (g) and several biogenic gases. (h) Receiver operating characteristic (ROC) curves of each gas in the diagnosis of colitis development. The cut-off value of the common logarithm (base 10) faecal lipocalin-2 level was 1.1. All the experiments were conducted three independent times.

Next, changes in the levels of biological gases during the induction of experimental colitis were examined. In DSS-treated mice, the H_2_ level initially increased transiently, decreased from day 4, declined further between days 7 and 8, and then gradually increased from day 8. Conversely, the H_2_ level in dextran-treated mice showed a similar transient peak on day 1 but continued fluctuating and exhibiting the same peak shape (Figure 3a). Likewise, the standard deviation of hydrogen concentration decreased gradually from day 1, bottomed out on days 6–8, and then began to increase in DSS-treated mice (Supplementary Figure 1a). In contrast, the standard deviation of H_2_ exhibited a periodical fluctuating and similar peak shape in dextran-treated mice (Supplementary Figure 1b). This trend was similar to the behaviour of the mean values, but the change is more evident for the standard deviation. In DSS-treated mice, the NH_3_ level remained high and stable until day 5 and then began to decrease. Following the discontinuation of DSS treatment after day 5, the NH_3_ level decreased rapidly. The NH_3_ level increased from day 8 and then continued to fluctuate with the decreased level. Contrastingly, in dextran-treated mice, NH_3_ levels increased until day 3 and remained stable (Figure 3b). Considering the changes in the levels of sulphur compounds after DSS treatment, H_2_S, CH_3_SH, and C_2_H_5_SH levels increased slowly from day 10 (Figure 3c–e). Additionally, CH_3_SH and C_2_H_5_SH levels declined between days 6 and 9 (Figure 3d,e). Regarding the level of sulphur compounds after dextran treatment, C_2_H_5_SH was stable with transient increases and decreases between days 2 and 5.

Further, the correlation between biological gases and colitis phenotypes, including changes in body weight and faecal LCN2 levels, were analysed. Given that the level of gases represented the sum of that in five mice, the mean level of gases per mouse for 24 h and the faecal level of LCN2 in each mouse were used to analyse the correlation. The H_2_ level was most negatively correlated with LCN2 levels and positively correlated with body weight (Figure 3f,g). CH_3_SH level was also negatively correlated with LCN2 levels (Figure 3g). In addition, the receiver operating characteristic (ROC) curve was plotted to assess the ability of each gas to detect the development of experimental colitis. The cut-off value of the common logarithm (base 10) faecal lipocalin-2 level was set as 1.1. ROC analysis showed that the area under the ROC curve (AUC) was high in H_2_S (AUC: 0.833) and H_2_ (AUC: 0.75), suggesting that these gases can be the predictive markers for the experimental colitis. Collectively, these results indicate that H_2_ is a strongly related gas for the outcome and development of DSS-induced colitis.

### *The relative abundance of Rikenellaceae and Akkermansiaceae is strongly correlated with the level of outcome of experimental colitis and H*_***2***_ ***level***

**Figure 4.**
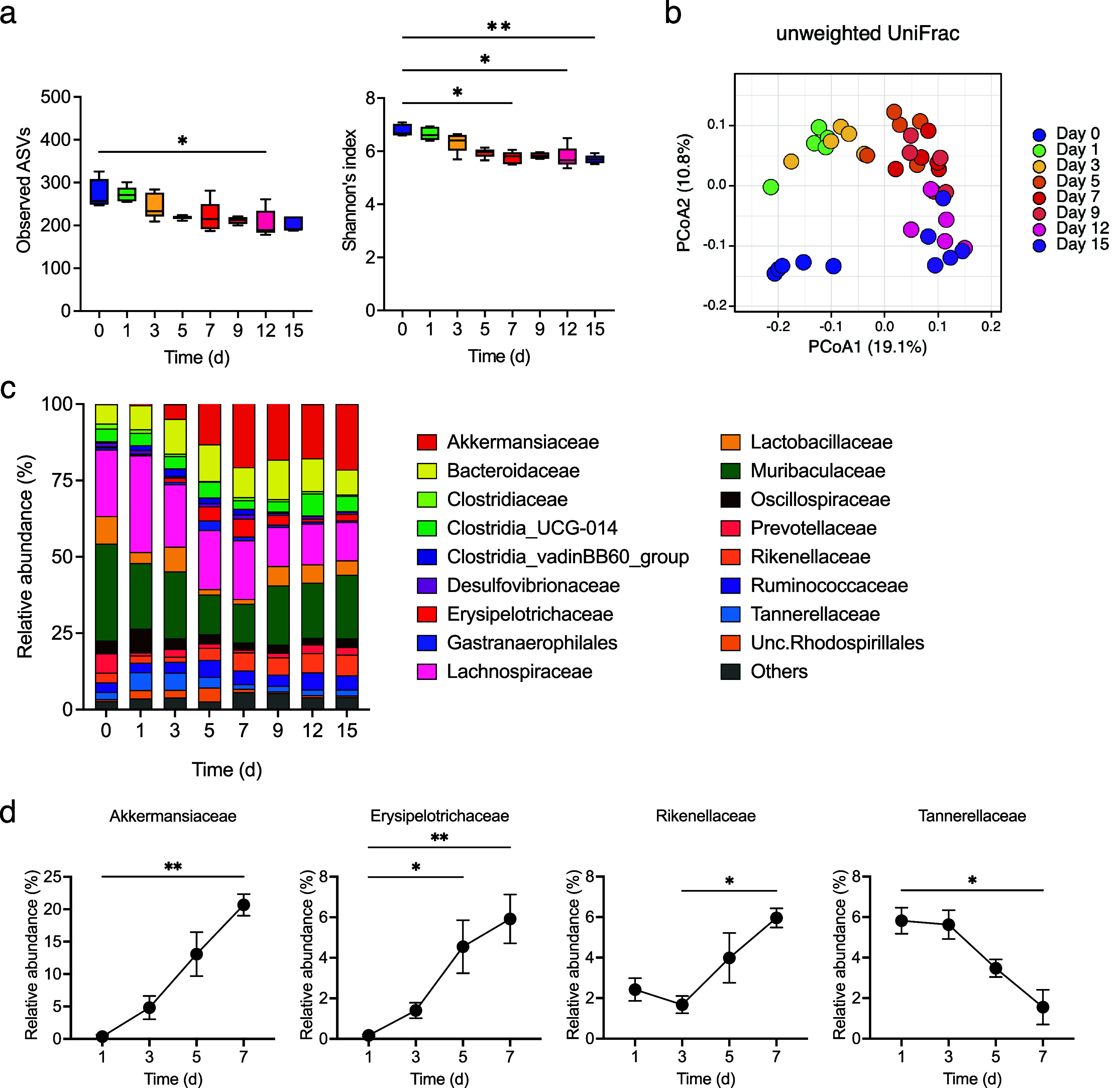
(a) Observed amplicon sequence variants (ASVs) (left panel) and α-diversity (right panel) for different time points, with box plots showing the median, interquartile range, and 1.5 interquartile range. (b) β-diversity in all samples at all points. Principal coordinates analysis (PCoA) of unweighted UniFrac distance matrix of different time points is shown, according to the legend in the figure. (c) Averaged relative abundance of ASVs in faecal samples from mice treated with DSS (n = 5). Analysis was performed on days 0, 1, 3, 5, 7, 9, 12 and 15 days. Colors correspond to each bacterial family. (d) Averaged relative abundance of faecal Akkermansiaceae, Erysipelotrichaceae, Rikenellaceae, and Tannerellaceae in DSS-treated mice on days 1, 3, 5, and 7. Friedman’s test was used in (a) and (d). * *p* < 0.05; ** *p* < 0.01; *** *p* < 0.001; ns., not significant. All the experiments were conducted three independent times.

Gut microbiota changes were examined, and the observed ASVs and α-diversity were found to decrease gradually during colitis development (Figure 4a). The β-diversity changed rapidly from day 0 to day 1 and underwent gradual alteration thereafter (Figure 4b). The relative abundance of Akkermansiaceae, Erysipelotrichaceae, and Rikenellaceae gradually increased from day 3 (at which the faecal LCN2 level started to increase) to day 7 (at which the faecal LCN2 level reached its peak), whereas that of Tannerellaceae decreased from day 3 to day 7 (Figure 4c,d).

**Figure 5.**
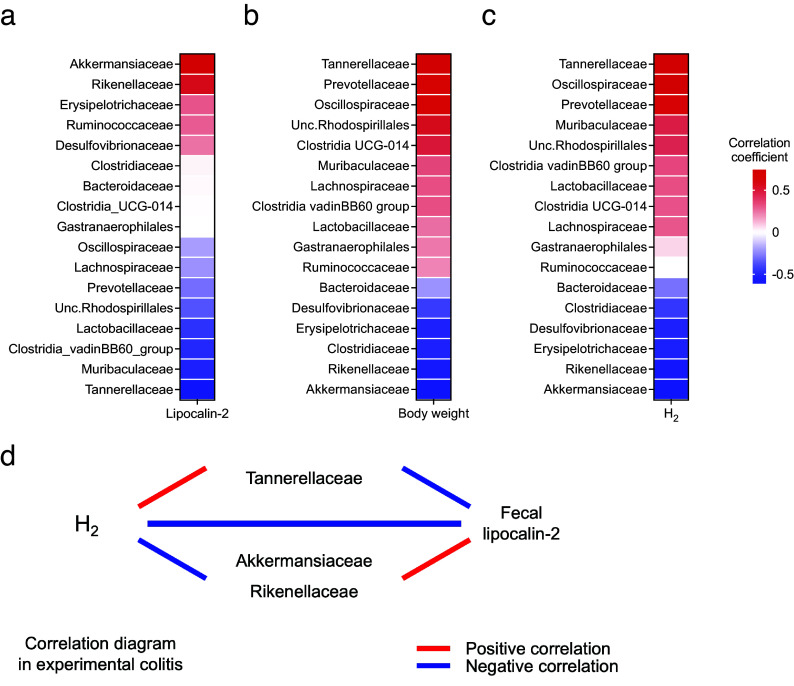
(a) Heatmap of the correlation between the average H_2_ levels and relative abundance of families of several intestinal microbes. (b–c) Heatmap of the correlation between the faecal LCN2 level or body weight change and relative abundance of families of several intestinal microbes. (d) Diagram of the relationship between H_2_ levels, LCN2 levels, and bacteria, which shows high or low correlation coefficients in the heatmap in (a–c).

Next, the correlation between changes in the gut microbiota and colitis phenotype and H_2_ levels was evaluated. The relative abundance of Akkermansiaceae and Rikenellaceae was positively correlated with LCN2 levels and negatively correlated with body weight changes. Conversely, the relative abundance of Tannerellaceae was negatively correlated with LCN2 levels and positively correlated with body weight changes (Figure 5a,b). Additionally, the relative abundance of Tannerellaceae and Oscillospiraceae was positively correlated with H_2_ levels; Tannerellaceae showed the most positive correlation with H_2_ levels. In contrast, the relative abundance of Akkermansiaceae and Rikenellaceae was negatively correlated with H_2_ levels (Figure 5c). These results indicate that the relative abundance of Tannerellaceae was positively correlated with the H_2_ level and body weight and negatively correlated with LCN2. However, the relative abundance of Akkermansiaceae and Rikenellaceae was negatively correlated with H_2_ levels and positively correlated with LCN2 levels (Figure 5d).

## Discussion

In this study, changes in five low-molecular-weight biogases, weight loss, faecal LCN2 levels, and gut microbiota composition during DSS-induced colitis were measured, and H_2_ level was found to have the strongest correlation with colitis outcomes. In addition, the relative abundance of certain gut bacteria was correlated with colitis pathology and H_2_ level changes.

Given that the DSS metabolism may, through the gut microbiota rather than the colitis phenotype, influence biogas levels, dextran was used as a negative control for the DSS treatment. A transient and rapid increase in H_2_ levels was observed immediately after administration of both DSS and dextran. Therefore, this change may be due to the gut microbiota consuming the dextran, which both compounds possess. The NH_3_ level continued to increase during both DSS and dextran administration and immediately declined every 24 h. This decrease may be due to the opening of the lid during measurement. Although the biogases in the cage were circulated through an air filter, NH_3_ may have accumulated inside the cage until the lid was opened (Salamonowicz et al., 2022). ROC analysis showed that the AUC was the highest in H_2_S. H_2_S is a gas produced by not only humans but also sulphate-reducing bacteria (SRB) in the gut (Barton et al., 2017). Indeed, higher activity of SRB and levels of H_2_S are found in stool samples from patients with UC (Levine et al., 1998; Medani et al., 2011). In addition to H_2_, therefore, H_2_S could be another biomarker to predict colitis development.

During the development of experimental colitis, the relative abundance of Tannerellaceae decreased proportionally as the severity of colitis increased, a trend consistent with reports showing a lower relative abundance of Tannerellaceae in patients with IBD (Alam et al., 2020). Furthermore, the abundance of Tannerellaceae was positively correlated with H_2_ levels. *Parabacteroides*, which belongs to the Tannerellaceae family, produces hydrogen (Ezeji et al., 2021). A recent study reported that increasing H_2_ levels in the gut relieved DSS-induced colitis symptoms by improving intestinal barrier function (Ge et al., 2022). In addition to H_2_ being a potential therapeutic agent for IBD, this study demonstrated its potential as a biomarker for IBD.

Furthermore, the relative abundance of Akkermansiaceae and Rikenellaceae increased with the severity of colitis and stabilised thereafter, although the relative abundance of Akkermansiaceae decreases in patients with IBD (Zhang et al., 2020; Zuo et al., 2022). However, in mouse studies, the association between Akkermansiaceae and colitis remains controversial. *Akkermansia* is involved in both inflammatory and anti-inflammatory effects (Jangi et al., 2016), such as Treg cell induction through short-chain fatty acid production or inflammatory responses by mucin degradation (Ganesh et al., 2013; Parada Venegas et al., 2019). An increase in the relative abundance of *Akkermansia* was observed with the exacerbation of colitis, indicating that the bacteria may be involved in colitis development. However, given the complex interaction between *Akkermansia* and colitis, it is necessary to analyse the gut microbiota and determine the characteristics of the bacteria in detail.

In this study, mouse models were used to continuously measure biogas components that were correlated to body weight, LCN2 levels, and gut microbiota composition from the onset to the symptom resolution of experimental colitis. However, this study was limited by its small sample size compared with that of clinical studies. Moreover, although the detection of exhaled breath components is desirable as a biomarker in clinical practice, it is important to note that the gas measured in this study was biogas, the components of which are an integrated product of various factors, including mouse exhaled breath, flatus, and faeces. All these factors are affected by the gut microbiota. Additionally, H_2_ and CO_2_, which are secondary metabolites of gut bacteria, are expelled from the body as exhaled breath and flatus and the metabolites and faeces strongly reflect gut microbiota changes (Zollner et al., 2021). These results suggest that DSS administration-induced changes in biogas components may be caused by the gut microbiota. Therefore, in the future, we will establish an experimental model capable of continuously measuring only the exhaled breath components.

In conclusion, this study established a negative correlation between the severity of experimental colitis and H_2_ levels, which were potentially mediated by gut bacteria. This study is valuable, as it focused on low-molecular-weight exhaled breath components that indicate the potential practical use of H_2_ as a biomarker for IBD.

## Materials and methods

### Mice

Specific pathogen-free C57BL/6 J male mice (6-week-old) were purchased from Sankyo Labo Service Corporation Inc. (Tokyo, Japan). All mice were housed at the Keio University Faculty of Pharmacy, Tokyo, and maintained on a normal regular chow diet (CE2; CLEA Japan, Inc., Tokyo, Japan) and tap water. Mice were subjected to a 12 h light/12 h dark cycle. The experiments were approved by the ethics committee of Keio University.

### Measurement of biogenic gas

The ventilation of the air flow line connected between the cage and the sensor gas chromatographs was performed using a diaphragm pump DAP-15 (ULVAC KIKO). H_2_ and NH_3_ concentrations were measured using a sensor gas chromatograph ODNA-P3-C (Nissha FIS). The sensor gas chromatograph comprised a gas sampler, gas column, and semiconductor gas sensor. The sampled gas was separated using the gas column, and highly selective gas detection was performed. The limit of detection (LOD) for H_2_ and NH_3_ was 1000 ppb. H_2_S, CH_3_SH, and C_2_H_5_SH concentrations were measured using the sensor gas chromatographs ODSA-P3-A (Nissha FIS). The LODs for H_2_S, CH_3_SH, and C_2_H_5_SH were 2 ppb, 5 ppb, and 5 ppb, respectively. The standard deviation of hydrogen concentration was also calculated.

### Treatment with DSS or dextran

DSS (MW: 36–50 kDa) (MP Biomedicals, Chiba, Japan) and dextran (TCI, Tokyo, Japan) were individually dissolved in distilled water at 2% (w/v), followed by filtration using Stericup (ø = 0.22 μm) (Merck, Darmstadt, Deutschland). All mice were administered 2% DSS or dextran solution for 5 days. Subsequently, on day 5, DSS or dextran was replaced with tap water. Body weight and food and water intake were monitored daily. To measure faecal LCN2 level and analyse faecal microbiota composition using 16S ribosomal RNA gene amplicon sequencing, faeces were obtained on days 0, 1, 3, 5, 7, 9, 12, and 15.

### Quantification of faecal lipocalin-2

The faecal LCN2 level was measured as a non-invasive intestinal inflammation biomarker (Chassaing et al., 2012). Mouse faecal pellets were collected in sterile 1.5 mL microcentrifuge tubes, and 100 mg/mL suspensions in sterile 0.1% (v/v) Tween-20/Dulbecco’s phosphate buffered saline (D-PBS) (−) were prepared. The samples were shaken using a vortex mixer at maximum speed for 20 min, followed by centrifugation at 15,000× *g* for 10 min. The supernatants were assayed for LCN2 using a mouse LCN2/NGAL DuoSet enzyme-linked immunosorbent assay (ELISA) (R&D Systems, Minneapolis, MN, USA) according to the manufacturer’s protocol.

### 16S rRNA gene analysis

Bacterial DNA was extracted from the mice faeces using the E.Z.N.A. Stool DNA Kit Pathogen Detection protocol (OMEGA) and purified using a magLEAD 12gC Automated Nucleic Acid Extraction System (Precision System Science Co., Ltd.). DNA samples were amplified by PCR using the following primers specific for the V3-V4 regions of the 16S rRNA gene: forward, 5-TCGTCGGCAGCGTCAGATGTGTATAAGAGACAGCCTACGGGNGGCWGCAG-3 and reverse, 5-GTCTCGTGGGCTCGGAGATGTGTATAAGAGACAGGACTACHVGGGTATCTAATCC-3′. Amplicon DNA was purified using AMPure XP beads (Beckman Coulter, Inc., Brea, CA, USA), and adapters were added by PCR using a Nextera XT index kit (Illumina, Inc., San Diego, CA, USA). Sequencing was performed using the MiSeq System (Illumina, Inc.). Sequencing data were analysed using QIIME2 version 2020.11 (Bolyen et al., 2019). The Cutadapt plugin in QIIME2 (https://doi.org/10.14806/ej.17.1.200, accessed on May 27, 2021) was used to trim the primer regions from the raw sequences. Sequences without primer regions were processed using the DADA2 algorithm (Callahan et al., 2016) to construct amplicon sequence variants (ASVs). BLAST (Camacho et al., 2009) was used to assign a taxonomy based on the SILVA database (version 138) (Pruesse et al., 2007), after randomly selecting high-quality sequence reads (11948) using a feature table (Weiss et al., 2017).

### Statistical methods

Statistical analyses were performed using GraphPad Prism software version 9.5.0 (GraphPad Software Inc.). Time-series data were analysed using the Shapiro–Wilk’s test to assess the normality of the data distribution, and all time-series data were non-normally distributed. Data were compared using Friedman’s test, followed by Dunn’s multiple comparison test to further determine the significance among groups. A p-value <0.05 was considered statistically significant. Observed ASVs was measured based on the actual number of distinct ASVs detected in a sample. α-diversity was measured by the Shannon’s diversity index that summarises both the species richness (total number of species) and evenness (abundance distribution across species) within a sample. For β-diversity analysis, dissimilarity between groups was calculated as unweighted UniFrac values for phylogenetic differences.

### Statistical analysis

The common logarithm (base 10) of faecal LCN2 levels was used as a non-invasive intestinal inflammation biomarker. H_2_ levels were averaged over 24 and 12 h before and after body weight measurement and faecal sample collection. Correlation analysis between body weight means and H_2_ levels and between LCN2 levels mean and H_2_ levels was performed using 6-point values, excluding days 0 and 1. Correlation analysis between body weight and LCN2 level means was performed on five samples with 6 points, excluding days 0 and 1, with 30 values. All correlation analyses were Pearson’s and were performed in R version 4.0.5 (2021-03-31) running under RStudio (2022.07.1 + 554). Receiver operating characteristic (ROC) curves were used for the diagnosis of colitis development, with 6-point values, excluding days 0 and 1 (days 3, 5, 7, 9, 12, and 15). The cut-off value of the common logarithm (base 10) faecal lipocalin-2 level was 1.1.

## Supporting information

Fujiki et al. supplementary material Fujiki et al. supplementary material

## Data Availability

All data reported in this paper will be shared by the corresponding authors upon request. This paper does not report original code.

## Supplementary material

The supplementary material for this article can be found at http://doi.org/10.1017/gmb.2023.17.
