# Supplementary material for: Hydrogen gas and the gut microbiota are potential biomarkers for the development of experimental colitis in mice
Source: Gut Microbiome (Camb). 2023 Nov 6;5:e3. doi: 10.1017/gmb.2023.17 (PMC11406375; doi:10.1017/gmb.2023.17)
Supplement: Fujiki et al. supplementary material [file S2632289723000178sup001.pdf]

Hydrogen gas and the gut microbiota are potential biomarkers for the development of experimental colitis in mice

Yuta Fujiki[a,b,d], Takahisa Tanaka[c,d], Kyosuke Yakabe[a,b], Natsumi Seki[a], Masahiro Akiyama[a], Ken Uchida[c,*], and Yun-Gi Kim[a,*]

[a]*Research Center for Drug Discovery, Faculty of Pharmacy and Graduate School of Pharmaceutical Sciences, Keio University, Tokyo 105-8512, Japan*

[b]*Division of Biochemistry, Faculty of Pharmacy and Graduate School of Pharmaceutical Sciences, Keio University, Tokyo 105-8512, Japan*

[c]*Department of Materials Engineering, The University of Tokyo, Tokyo 113-8656, Japan*

[d] These authors contributed equally to this work

*Corresponding authors: ykim@keio.jp, uchidak@material.t.u-tokyo.ac.jp

a

b

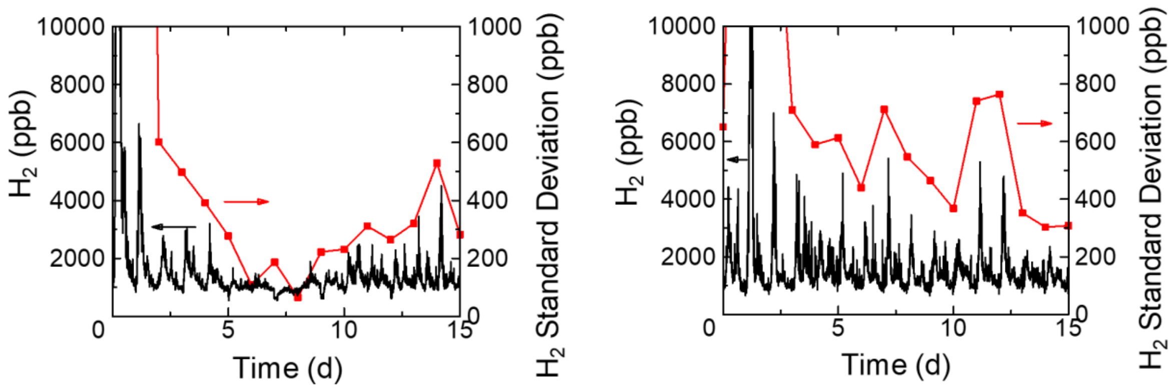

Supplementary Figure 1. H_2 fluctuation correlates with intestinal inflammation. H_2 gas was detected using the instrument introduced in Figure 1 for 15 days. The kinetics (black line) and the standard deviation (red line) of H_2 concentration for 15 days in DSS-treated (a) and dextran-treated mice (b).
